# Fiber Optic Gyro Random Error Suppression Based on Dual Adaptive Kalman Filter

**DOI:** 10.3390/mi16080884

**Published:** 2025-07-29

**Authors:** Hongcai Li, Zhe Liang, Zhaofa Zhou, Zhili Zhang, Junyang Zhao, Longjie Tian

**Affiliations:** 1 Intelligent Control Laboratory, PLA Rocket Force University of Engineering, Xi’an 710025, China; 2 Institute of Optics and Electronics, School of Instrumentation Science and Optoelectronics Engineering, Beihang University, Beijing 100191, China

**Keywords:** fiber optic gyroscope, random error identification, time series model, Kalman filter, inertial information measurement, random error modeling

## Abstract

The random error of fiber optic gyros is a critical factor affecting their measurement accuracy. However, the statistical characteristics of these errors exhibit time-varying properties, which degrade model fidelity and consequently impair the performance of random error suppression algorithms. To address these issues, this study first proposes a recursive dynamic Allan variance calculation method that effectively mitigates the poor real-time performance and spectral leakage inherent in conventional dynamic Allan variance techniques. Subsequently, the recursive dynamic Allan variance is integrated with the process variance estimation of Kalman filtering to construct a dual-adaptive Kalman filter capable of autonomously switching and adjusting between model parameters and noise variance. Finally, both static and dynamic validation experiments were conducted to evaluate the proposed method. The experimental results demonstrate that, compared to existing algorithms, the proposed approach significantly enhances the suppression of angular random walk errors in fiber optic gyros.

## 1. Introduction

The fiber-optic gyroscope (FOG) serves as the core sensing element in strapdown inertial navigation systems (SINS). Compared to ring laser gyroscopes (RLGs), FOGs exhibit significant advantages, including all-solid-state construction, low cost, and low noise. Among FOG technologies, the interferometric fiber-optic gyroscope (IFOG) has reached a mature stage of development and is currently the mainstream attitude measurement component in fiber-optic SINS [1,2]. However, due to the working principle of IFOGs, environmental factors such as temperature and vibration can significantly affect their output signals, introducing measurement errors known as gyro drift. IFOG drift is categorized into systematic drift and random drift. While the former can be compensated through calibration, the latter has become the most critical metric for evaluating IFOG accuracy. Therefore, accurate modeling and effective suppression of IFOG random errors are of substantial engineering significance [3,4,5].

Accurate modeling of IFOG random errors is a critical prerequisite for their effective suppression. Currently, mainstream modeling approaches focus on fitting methods represented by the autoregressive moving average (ARMA) model and decomposition-reconstruction methods such as empirical mode decomposition (EMD) and wavelet transform (WT), which have been extensively studied. The ARMA model, a widely used tool in time series analysis, has been applied to IFOG random error suppression. Narasimhappa et al. proposed an adaptive robust Kalman filter based on the AR model, which constrained filter divergence through innovation sequence covariance, achieving notable noise suppression. However, the modeling process was not detailed [6]. Jin et al. introduced an adaptive Kalman filtering method based on covariance matching, improving the AR model’s mean-following capability. Yet, it did not account for the time-varying statistical characteristics of IFOG data [7]. Song et al. proposed another AR model-based adaptive Kalman filtering method but similarly overlooked the real-time variation in IFOG data statistics [8].

EMD and WT methods have also been widely applied to gyro random error modeling. Shu et al. employed three EMD-based filtering methods for IFOG random drift, demonstrating superior error suppression compared to other algorithms [9]. Wang et al. proposed an improved EMD method combined with recursive least squares denoising, showing better performance than traditional EMD methods [10]. Gao et al. developed a variational mode decomposition (VMD)-based denoising algorithm for IFOG signals, validating its effectiveness [11]. Ma et al. designed an optimized wavelet filter for gyro random error suppression, determining the optimal wavelet decomposition level through extensive testing [12]. While EMD and WT methods excel in certain aspects, they struggle with accurate modeling of low-frequency noise (long correlation time errors) and require large datasets, resulting in poor real-time performance. In contrast, the ARMA model, when used as the system state equation in Kalman filtering, demonstrates superior filtering effectiveness and real-time performance. Consequently, ARMA model-based IFOG random error modeling and suppression have become the dominant research direction in inertial navigation. Existing modeling methods typically fit ARMA model parameters using limited training data and apply the model throughout the entire error suppression cycle. However, the statistical characteristics of IFOG random errors vary over time, degrading model accuracy and impairing the performance of suppression algorithms.

The dynamic Allan variance (DAVAR) method, compared to traditional Allan variance, effectively reflects the dynamic stability of IFOG random errors and is commonly used for testing and analyzing IFOG dynamic performance. However, its computational real-time performance is hindered by extensive data reuse and repetitive calculations [13]. Additionally, the truncation of IFOG data by window functions leads to spectral leakage [14]. Researchers have explored adaptive adjustments to window function length and type [15] and methods to reduce computational complexity [16]. Nonetheless, these approaches remain essentially offline processing methods for IFOG data.

To address the aforementioned issues, this paper first proposes a recursive Allan variance (RAVAR) calculation method based on the characteristics of the Allan variance formula. This method is further extended to DAVAR computation, significantly improving computational efficiency and mitigating the spectral leakage problem. Subsequently, a **dual-adaptive Kalman filter (DAKF)** is constructed based on the recursive DAVAR, which enables simultaneous automatic switching and adjustment of model parameters and variance information. This approach aims to more accurately identify and correct the factors causing dynamic characteristic changes in FOG data. Finally, the effectiveness of the proposed method is validated through experiments on FOG random error suppression.

## 2. A Recursive Dynamic Allan Variance Calculation

### 2.1. Allan Variance Calculation

The random errors in IFOG output include quantized noise (QN), angle random walk (ARW), bias instability (BI), angular rate random walk (RRW), rate ramp (RR), and other noise sources [17]. These errors exhibit the power law noise characteristics. Allan variance, as a widely used time-domain analysis method, can identify the source of a given noise in the data, so it is often used to determine the noise components of IFOG signal. In most cases, different noise terms appear in different cluster time τ area, so various random processes present in the observed data can be readily identified and characterized. The variance associated with IFOG random errors is typically expressed relative to the angular rate output level. Based on the definition of Allan variance, its calculation process involves the following steps [18]

(1) $$\sigma^{2}(\tau )=\frac{1}{2(k-1)}\sum_{i=1}^{k}{\left({\bar{\Omega }}_{i+1}-{\bar{\Omega }}_{i}\right)}^{2}$$

(2) $${\bar{\Omega }}_{i}=\sum_{j=i}^{i+m-1}\Omega_{j}/m,k=\left\lfloor \frac{N}{m}\right\rfloor ,\tau =m\tau_{0},\;m=1,2,\ldots ,\left\lfloor \frac{N}{2}\right\rfloor$$

Among them, $\sigma^{2}(\tau )$ is the Allan variance corresponding to different cluster times τ, $\Omega_{j}$ is the sampling value at the time $t_{j}$ when the IFOG angular rate is output, N is the total amount of IFOG output data, k is the number of cluster intervals, and m is the number of data within each cluster interval. ${\bar{\Omega }}_{i}$ is the mean of the angular rate in each cluster time, $\tau_{0}$ is the sampling time, and ⌊ ⌋ represents the downward rounding operation.

There is the following quantitative relationship between the Allan variance of FOG random noise and the power spectral density [19]

(3) $$\sigma^{2}(\tau )=4\int_{0}^{\infty }S_{\Omega }(f)\frac{\sin^{4}(\pi f\tau )}{{\left(\pi f\tau \right)}^{2}}df$$

In Formula (3), $S_{\Omega }(f)$ is the power spectral density function and f is the spectrum variable of noise. In the time-domain, various error source characteristic parameters of IFOG can be obtained from its output data.

### 2.2. Dynamic Allan Variance Calculation

The DAVAR method represents an extension of the traditional Allan variance technique, specifically designed to quantify the instantaneous stability characteristics of inertial devices [20,21]. While the conventional Allan variance provides a two-dimensional characterization of random error processes. It is limited in that it can only assess the stationarity of the output data from the inertial device. In contrast, the DAVAR method offers a three-dimensional manifestation that can assess the non-stationary changes in random errors. In the DAVAR method, data are intercepted for Allan variance estimation by a rectangular window with a length T at the given time $\;t_{1}$ as the center point. Then, the window slides to the next center moment $t_{2}$, and estimates the Allan variance of the rectangular window data at this moment. It should be noted that rectangular windows at two adjacent moments must have overlapping parts.

Considering continuous time data measurement, assuming $x(t_{j})$ is the random error data measured by the FOG, a rectangular window $P_{T}(t^{\prime })$ with a window interval 1−T/2≤t′≤1+T/2 to intercept the random error data $x(t^{\prime })$ could be used, and the obtained data are

(4) $$x_{T}(t_{j},t^{\prime })=x(t^{\prime })P_{T}(t_{j}-t^{\prime })$$

In Equation (4), $x_{T}(t_{j},t^{\prime })$ is the intercepted data. A rectangular window $P_{T}(t)$ of length T is defined in the following form

(5) $$P_{T}(t_{j})=\left\{\begin{array}{c} \;1,\left|t_{j}\right|⩽T/2\\ 0,Others \end{array}\right.$$

For any window, time $t_{j}$ is a fixed parameter, which represents the center of window $P_{T}(t_{j}-t^{\prime })$. $t^{\prime }$ represents the time lost in the window. An incremental process $\Delta (t,t^{\prime },\tau )$ can be established by performing convolution operations through the intercepted data and Allan variance estimation window $A_{\tau }(t^{\prime })$

(6) $$\Delta (t,t^{\prime },\tau )=\int_{-\infty }^{+\infty }A_{\tau }(t^{\prime }-t^{\prime \prime })x_{T}(t,t^{\prime \prime })dt^{\prime \prime }$$

The constraints of each variable in the formula are as follows

(7) $$A_{\tau }(t^{\prime })=\left\{\begin{array}{c} -\frac{1}{\tau },\;0⩽t^{\prime }<\tau \\ \frac{1}{\tau },\;-\tau ⩽t^{\prime }<0 \end{array}\right.$$

In Equation (7), $t-(T/2-\tau )⩽t^{\prime }⩽t+(T/2-\tau )$, $0<\tau ⩽\tau_{\max}$, $\tau_{\max}$ is the largest observation time interval of data in the Allan variance estimation window. Substitute the incremental process in Equation (7) into Equation (1), and the dynamic Allan variance corresponding to different moments $t_{j}$ can be obtained.

### 2.3. A Recursive Dynamic Allan Variance Calculation

Through Formula (1), it can be rewritten into the following form

(8) $$\sigma_{k}^{2}(\tau )=\frac{1}{2(k-1)}\sum_{i=2}^{k-1}[{\left({\bar{\Omega }}_{i}-{\bar{\Omega }}_{i-1}\right)}^{2}+{\left({\bar{\Omega }}_{k}-{\bar{\Omega }}_{k-1}\right)}^{2}]$$

Further, it can be derived

(9) $$\begin{array}{l} \sigma_{k}^{2}(\tau )=\frac{k-2}{k-1}[\frac{1}{2(k-2)}\sum_{i=2}^{k-1}{\left({\bar{\Omega }}_{i}-{\bar{\Omega }}_{i-1}\right)}^{2}]+\frac{1}{2(k-1)}{\left({\bar{\Omega }}_{k}-{\bar{\Omega }}_{k-1}\right)}^{2}\\ =(1-\frac{1}{k-1})\sigma_{k-1}^{2}(\tau )+\frac{1}{2(k-1)}{\left({\bar{\Omega }}_{k}-{\bar{\Omega }}_{k-1}\right)}^{2} \end{array}$$

Formula (9) is the recursive calculation form of Allan variance, which can realize the online calculation of Allan variance of IFOG output data, which can greatly improve the shortcomings of poor real-time performance of Allan variance calculation.

**Figure 1:**
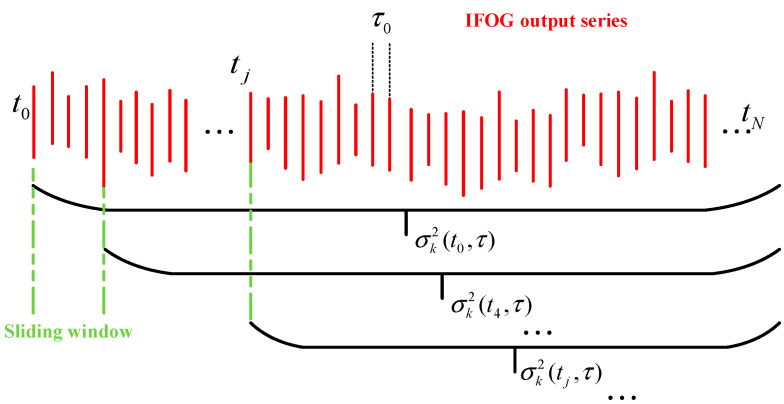
Schematic diagram of recursive DAVAR calculation process.

Similarly, as demonstrated in prior work, the dynamic Allan variance principle is actually used to identify the error characteristics of IFOG data within a limited range on both sides at a certain moment through the sliding window. Referring to the idea of dynamic Allan variance, we can introduce the idea of time sliding into the calculation of recursive Allan variance and slide the starting data point of recursive Allan variance. First, starting from the initial time $\;t_{0}$ of the FOG’s working cycle, the first set of Allan variances can be recursively calculated based on Formula (9), and then the output corresponding to the FOG at the working time $\;t_{1}$ is selected as the starting data point for a period of time, and the same is based on Formula (9) recursively calculates the second set of Allan variances, and so on. Multiple sets of Allan variance curves can be obtained at different moments $t_{j}$. The specific calculation process diagram is shown in Figure 1.

In Figure 1, $\sigma_{k}^{2}(t_{j},\tau )$ represents the Allan variance corresponding to the starting data point with the time $t_{j}$ as the starting data point. It can also be seen from Figure 1 that there are overlapping parts in the corresponding Allan variance solution interval at each time, and at the same time, each interval will be extended infinitely over time, and there is no operation of artificial intercepting data, which can greatly improve the power leakage problem caused by data truncation. In addition, Formula (9) can also find that the recursiveness has the characteristic of gradually disappearing memory, that is, as k increases, the weight occupied by the corresponding angular rate difference square ${\left({\bar{\Omega }}_{k}-{\bar{\Omega }}_{k-1}\right)}^{2}$ decreases, which ensures that each group of Allan variance can retain long correlation error information and accurately reflect the discrete situation of FOG data near time $t_{j}$.

## 3. Design of Kalman Filter Based on ARMA Model

The use of Kalman filtering based on the ARMA model to suppress the random error of FOG has obvious advantages under the high real-time requirements of inertial navigation scenarios [22,23]. The ARMA model is a composite form of AR model and MA model. It can generally be used alone or in combination according to actual needs. At the same time, these three models can also be transformed into each other [24]. The general form of the ARMA model is as follows

(10) $$A\left(B^{-1}\right)x\left(t\right)=C\left(B^{-1}\right)\varepsilon \left(t\right)$$

At the same time, there are definitions like

(11) $$A\left(B^{-1}\right)=1-\sum_{i=1}^{p}a_{i}B^{-i},\;C\left(B^{-1}\right)=1+\sum_{i=1}^{q}c_{i}B^{-i}$$

In Equation (11), $a_{i}$, $c_{i}$ are model coefficients, p,q are model orders, B is delay operator (e.g., $B^{-i}x(t_{j})=x(t_{j-i})$), and $\varepsilon \left(t_{j}\right)$ are white noise sequences, where the expression of the AR model is

(12) $$A\left(B^{-1}\right)x\left(t_{j}\right)=\varepsilon \left(t_{j}\right)$$

**Figure 2:**
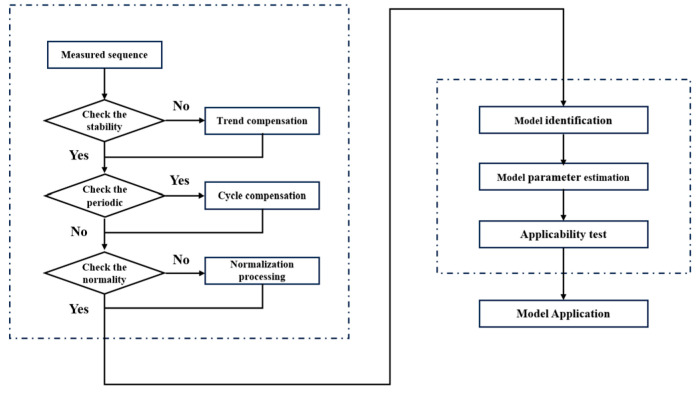
Schematic diagram of ARMA model modeling process.

Observing Formula (12), it can be seen that the AR model has the characteristics of discrete superposition of time-related terms and white noise terms, which is consistent with the data characteristics of FOGs. Therefore, this model has become a common model for suppressing random walk errors in FOG angles. The specific modeling process is shown in Figure 2 and is not described here.

The determination of model order is the key to affect modeling accuracy. At present, the commonly used model order determination is based on AIC criterion or BIC criterion [25]. Taking AIC criterion as an example, its measurement index can be given by the following formula

(13) $$AIC=2n_{c}+N_{t}(\sigma_{Mod}^{2})$$

In Formula (13), $n_{c}$ is the number of parameters of the optimal model, $N_{t}$ is the number of training samples, and $\sigma_{Mod}^{2}$ is the fitted residual variance (MSE) of the AR model, which conforms to the white noise characteristics.

Based on the IFOG random error model obtained by the above theory, the Kalman filter is constructed, and the discrete state space model is as follows

(14) $$\left\{\begin{array}{l} X_{j}=\Phi_{j,j-1}X_{j-1}+BW_{j}\\ Z_{j}=H_{j}X_{j}+V_{j} \end{array}\right.$$

In Equation (14), $X_{j}$ is the state vector at time $t_{j}$, $\Phi_{j,j-1}$ is the state transition matrix, $W_{j}$ is the system noise vector at time $t_{j}$, B is the system noise figure matrix, $Z_{j}$ is the FOG output observation value at time $t_{j}$, $H_{j}$ is the measurement matrix, and $V_{j}$ is the observation noise at time $t_{j}$, where the statistical properties of $W_{j}$ and $V_{j}$ are as follows

(15) $$E[W_{j}]=0,E[V_{j}]=0,E[W_{j}W_{l}^{T}]=Q_{j},E[V_{j}V_{l}^{T}]=R_{j},E[W_{j}V_{l}^{T}]=0$$

Wherein $Q_{j}$ is the system noise variance and $R_{j}$ is the measurement noise variance at time $t_{j}$.

For example, if the stochastic error model of the FOG is

(16) $$x_{j}=a_{1}x_{j-1}+a_{2}x_{j-2}+a_{3}x_{j-3}+\varepsilon$$

select the state variables and system output as

(17) $$X_{j-1}={\left[\begin{array}{ccc} x_{j-1} & x_{j-2} & x_{j-3} \end{array}\right]}^{T},Z_{j}=x_{j}$$

then the state space model parameters of FOG can be written as

(18) $$\Phi_{j,j-1}=\left[\begin{array}{ccc} a_{1} & a_{2} & a_{3}\\ 1 & 0 & 0\\ 0 & 1 & 0 \end{array}\right],\;B=\left[\begin{array}{ccc} 1 & 0 & 0\\ 0 & 0 & 0\\ 0 & 0 & 0 \end{array}\right],\;H_{j}=\left[\begin{array}{ccc} 1 & 0 & 0 \end{array}\right]$$

## 4. An Improved Kalman Filter Based on Dual Adaptive Mechanism

Kalman filtering requires that system noise and measurement noise are white noise sequences of known orders, but in fact, the random time-degeneration of FOG drift will greatly reduce the accuracy of this filtering. In view of the uncertainty of noise statistical characteristics, Sage–Husa adaptive filter adds a time-varying noise estimator to the KF algorithm framework, realizing adaptive estimation of noise variance [26]. However, in actual situations, Sage–Husa filtering will easily cause filter abnormalities due to strong coupling of $Q_{j}$ and $R_{j}$ during the estimation process. Adaptive Kalman filtering based on covariance matching can effectively avoid the occurrence of filter abnormalities [27,28], the following discrimination conditions are introduced

(19) $$v_{j}^{T}\nu_{j}>\lambda tr(E[\nu_{j}v_{j}^{T}])\Rightarrow \nu_{j}v_{j}^{T}>H_{j}P_{j,j-1}H_{j}^{T}+R_{j}$$

wherein λ≥1 is the reserve coefficient, $\nu_{j}$ is the filter remainder at time $t_{j}$, $v_{j}^{T}\nu_{j}$ is the actual filter remainder variance matrix at time $t_{j}$, $P_{j,j-1}$ is the one-step prediction mean square error matrix from time $t_{j-1}$ to time $t_{j}$ In the filtering process, the state of the filtering is judged by using the Equation (19). If the Equation (19) is established, it means that there is an abnormal filtering, and the original noise information is no longer suitable for the current filtering. At this time, the current observation data information should be used to re-estimate $Q_{j}$ and $R_{j}$ to make them adapt to the current filtering. If the Equation (19) is not established, it means that there is no anomaly in the filtering, and there is no need to re-estimate $Q_{j}$ and $R_{j}$.

As observed, the aforementioned adaptive Kalman filters operate by adjusting their performance based on estimation outcomes and are designed to solely address the variance information of measurement white noise and process white noise. However, in dynamic systems, if there is severe distortion in the system model, the resulting error is composed of error terms such as white noise, colored noise, periodic error and trend term error, and does not conform to the single white noise feature, then forcibly adaptively correcting the variance information of white noise will lead to limited filtering accuracy or distortion of the filtering result. Therefore, it is necessary to design a dual adaptive Kalman filter that can automatically switch model parameters and variance information at the same time, which will affect the factors that cause filtering abnormalities during the Kalman filtering process (mainly the changes in model accuracy and the measurement of noise variance).

It is known that the variance calculation of the Allan variance mentioned above can reflect the real-time dynamic stability of the FOG. Therefore, the Allan variance can be introduced into the adaptive Kalman filter and its variance estimation result is used as the outside world. The constraints of the Allan variance are used to determine which type of influencing factors are corrected.

The specific idea is: based on the real-time output sequence of the same group of FOG, the state Allan variance algorithm and the adaptive Kalman filtering algorithm are used to perform parallel operations, and comparing the dynamic Allan variance $\sigma_{k}^{2}(t_{j},\tau_{0})$ at the current moment $t_{j}$ and the dynamic Allan variance $\sigma_{k}^{2}(t_{j-1},\tau_{0})$ at the previous moment. If $\sigma_{k}^{2}(t_{j},\tau_{0})\geq \sigma_{k}^{2}(t_{j-1},\tau_{0})$, it means that the variance of the measured noise of the system is increasing. At this time, attention should be paid to judge the measured noise estimate ${\overset{\wedge }{R}}_{j}$ during the filtering process. If the magnitudes of the values of ${\overset{\wedge }{R}}_{j}$ and $\sigma_{k}^{2}(t_{j},\tau_{0})$ are basically the same, it means that the filtering effect is within the expected and the filtering is carried out normally. If there is a relatively large error between the values of ${\overset{\wedge }{R}}_{j}$ and $\sigma_{k}^{2}(t_{j},\tau_{0})$, it means that the variance estimation accuracy during the filtering process becomes worse. At this time, $\sigma_{k}^{2}(t_{j},\tau_{0})$ should be used to replace ${\overset{\wedge }{R}}_{j}$. If $\sigma_{k}^{2}(t_{j},\tau_{0})<\sigma_{k}^{2}(t_{j-1},\tau_{0})$, it means that the variance of the measured noise of the system is decreasing. At this time, the AR model established before may be overfitted, so the model should be re-established based on the data sequence near the current moment.

The flow of the dual-adaptive Kalman filtering algorithm is as follows

*a.*  Build an AR model based on AIC criteria [29];
*b.*  Setting initial parameters for filtering
  (20) $$X_{0},P_{0},r_{0},R_{0},q_{0},Q_{0},B,j=1$$
*c.*  Updating the adaptive variance weight coefficients
  (21) $$d_{j}=(1-b)/(1-b^{j+1})$$
*d.*  Recursively calculate the dynamic Allan variance $\sigma_{k}^{2}(t_{j-1},\tau_{0})$ at time $t_{j}$ based on Formula (9);
*e.*  One-step prediction of filters
  (22) $${\hat{X}}_{j,j-1}=\Phi_{j,j-1}{\hat{X}}_{j-1}+{\hat{q}}_{j-1}$$
  (23) $$P_{j,j-1}=\Phi_{j,j-1}P_{j-1}\Phi_{j,j-1}^{T}+{\hat{Q}}_{j-1}$$
*f.*  Calculating filter remainder
  (24) $${\hat{r}}_{j}=(1-d_{j}){\hat{r}}_{j-1}+d_{j}(Z_{j}-H_{j}{\hat{X}}_{j,j-1})$$
  (25) $$\nu_{j}=Z_{j}-H_{j}{\hat{X}}_{j,j-1}-{\hat{r}}_{j}$$
*g.*  Judging the relationship between $\sigma_{k}^{2}(t_{j},\tau_{0})$ and $\sigma_{k}^{2}(t_{j-1},\tau_{0})$. If $\sigma_{k}^{2}(t_{j},\tau_{0})\geq \sigma_{k}^{2}(t_{j-1},\tau_{0})$, proceed to step *h*. If $\sigma_{k}^{2}(t_{j},\tau_{0})<\sigma_{k}^{2}(t_{j-1},\tau_{0})$, then go back to step *a* to remodel based on the data sequence near the current moment;
*h.*  Judging the relationship between $\sigma_{k}^{2}(t_{j},\tau_{0})$ and ${\overset{\wedge }{R}}_{j}$. If the magnitudes of the values of ${\overset{\wedge }{R}}_{j}$ and $\sigma_{k}^{2}(t_{j},\tau_{0})$ are basically the same, continue with step *i*. If a relatively large error occurs between the values of ${\overset{\wedge }{R}}_{j}$ and $\sigma_{k}^{2}(t_{j},\tau_{0})$, then ${\overset{\wedge }{R}}_{j-1}=\sigma_{k}^{2}(t_{j},\tau_{0})$ and then proceed to step *i*;
*i.*  Estimate noise measurement
  (26) $${\hat{R}}_{j}=(1-d_{j}){\hat{R}}_{j-1}+d_{j}\left[\nu_{j}\nu_{j}^{T}-H_{j}P_{j,j-1}H_{j}^{T}\right]$$
*j.*  Filter gain update
  (27) $$K_{j}=P_{j,j-1}H_{j}^{T}{\left[H_{j}P_{j,j-1}H_{j}^{T}+{\hat{R}}_{j}\right]}^{-1}$$
*k.*  Optimal estimate
  (28) $${\hat{X}}_{j}={\hat{X}}_{j,j-1}+K_{j}[Z_{j}-H_{j}{\hat{X}}_{j,j-1}]$$
*l.*  Mean square deviation update
  (29) $$P_{j}=[I-K_{j}H_{j}]P_{j,j-1}$$
*m.*  Calculation process noise
  (30) $${\hat{q}}_{j}=(1-d_{j}){\hat{q}}_{j-1}+d_{j}\left({\hat{X}}_{j}-\Phi_{j,j-1}{\hat{X}}_{j-1}\right)$$
  (31) $${\hat{Q}}_{j}=(1-d_{j}){\hat{Q}}_{j-1}+d_{j}\left(K_{j}\nu_{j}\nu_{j}^{T}K_{j}^{T}+P_{j}-\Phi_{j,j-1}P_{j-1}\Phi_{j,j-1}^{T}\right)$$

In Equations (20)–(31), $P_{j}$ is the prediction mean square error matrix at time $t_{j}$, $q_{j}$ is the system noise mean at time $t_{j}$, and $r_{j}$ is the measurement noise mean at time $t_{j}$, $q_{j}$ and $r_{j}$ are generally 0, $d_{j}=(1-b)/(1-b^{j+1})$ is the variance weight coefficient of adaptive filtering, the value range of b is generally [0.95,0.99], and the rest of the variables are consistent with the previous definitions.

## 5. Experimental Verification

**Figure 3:**
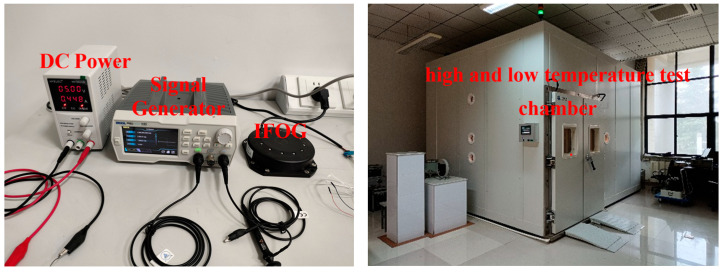
FOG random error suppression experimental equipment.

To verify the effectiveness of the dual adaptive Kalman filter mentioned in this article, random errors in the IFOG output sequence are suppressed. First, select three 120 IFOGs, marked as IFOG-A, IFOG-B, and IFOG-C; then, collect static data for 5 h for each IFOG, at 1 Hz, and select the short-term data for the first 30 min. Model the random error; finally, based on the dual adaptive Kalman filter (denoted as DSHKF) proposed in this paper, ARW error suppression processing is performed on three groups of IFOG data. In addition, the experimental results of ARW error filtering by adaptive Kalman filter (VSHKF) and classical Kalman filter (CKF) based on covariance matching are taken as the control experimental group, and the Allan variance is uniformly carried out after filtering. The equipment used for the experiment of random error suppression of fiber optic gyroscopes is shown in Figure 3 (from left to right are fiber optic gyroscope, high and low temperature test chamber). The model information of the control experimental group is shown in Table 1.

**Figure 4:**
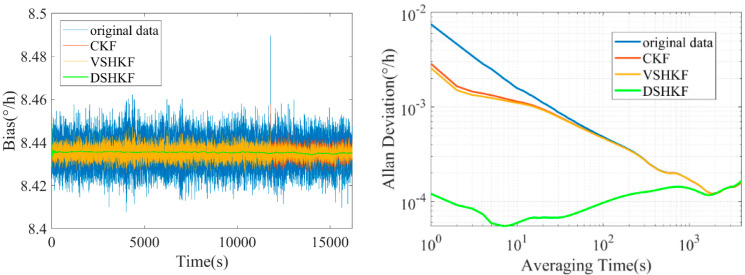
IFOG−A output filtering results and Allan variance curves at room temperature.

**Figure 5:**
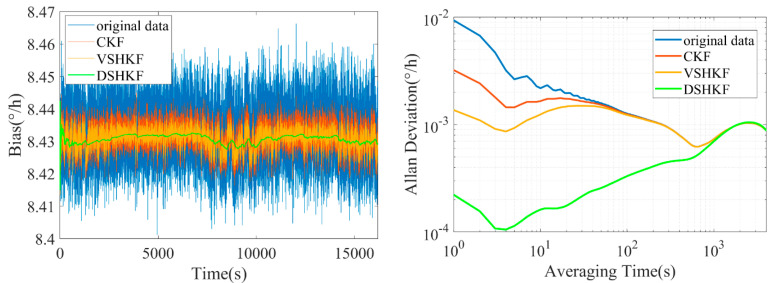
IFOG−B output filtering results and Allan variance curves at room temperature.

**Figure 6:**
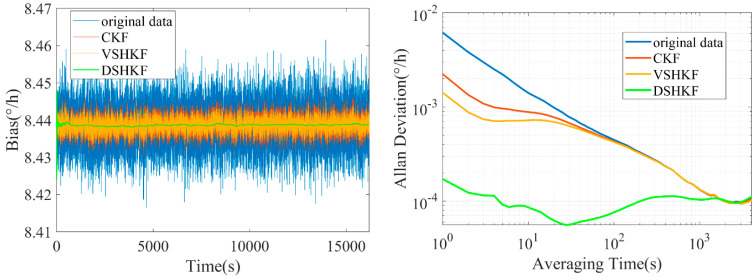
IFOG−C output filtering results and Allan variance curves at room temperature.

The model in Table 1 is substituted into CKF and VSHKF as the system state equations respectively to suppress the random errors in the three IFOG output sequences. The output sequence before and after filtering through each algorithm and its corresponding Allan variance curve are shown in Figure 4, Figure 5 and Figure 6, respectively.

The power spectrum coefficients of the random walking error of the angle of IFOG before and after filtering are counted. The statistical results are shown in Table 2.

According to the data in Table 2, the DSHKF algorithm suppresses the ARW error of the three IFOGs. For IFOG-A, the ARW coefficient of the filtered data after DSHKF algorithm is reduced by 97.12%, 93.87%, and 92.90% respectively compared with the original data, the filtered data after CKF algorithm, and the filtered data after VSHKF algorithm. For IFOG-B, the ARW coefficient of the filtered data after DSHKF algorithm is reduced by 97.19%, 92.00%, and 85.30% respectively compared with the original data, the filtered data after CKF algorithm, and the filtered data after VSHKF algorithm. For IFOG-C, the ARW coefficient of the filtered data after DSHKF algorithm is reduced by 96.22%, 89.96%, and 85.00% respectively compared with the original data. The CKF algorithm has the worst filtering effect.

**Figure 7:**
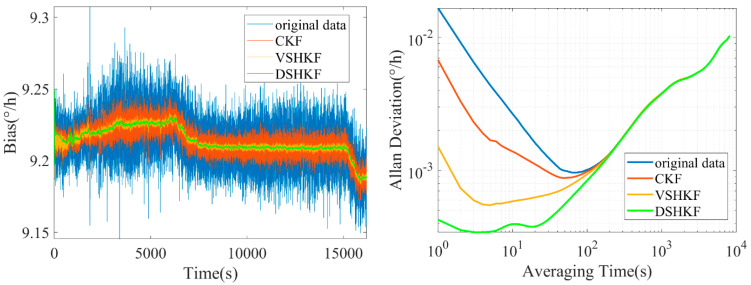
IFOG output filtering results and Allan variance curves under variable temperature conditions.

Further, one IFOG is arbitrarily selected among the above three IFOGs, and a random error suppression test is carried out on the IFOG output data under variable temperature, so as to test the dynamic performance of the DSHKF algorithm. The temperature change rate is set to 1 °C/min, and the 5 h variable temperature data are collected. The temperature data are 1 Hz. The short-term data of the first 30 min are selected as the modeling of random errors. The rest of the experimental process is consistent with the normal temperature experiment, and the experimental results are shown in Figure 7.

**Figure 8:**
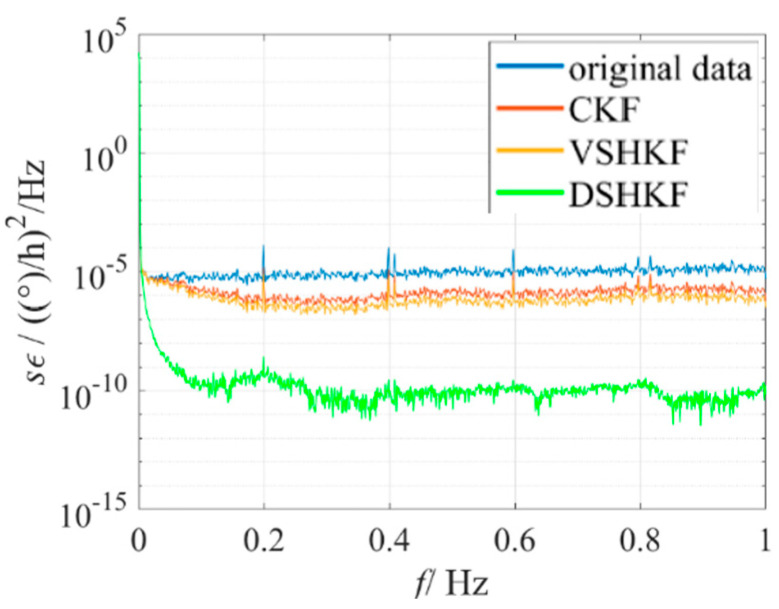
IFOG−A’s PSD at room temperature.

**Figure 9:**
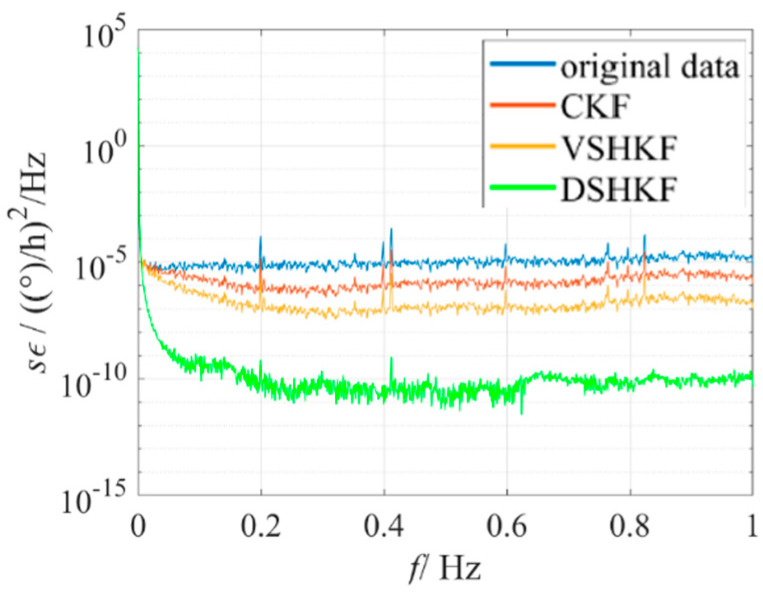
IFOG−B’s PSD at room temperature.

**Figure 10:**
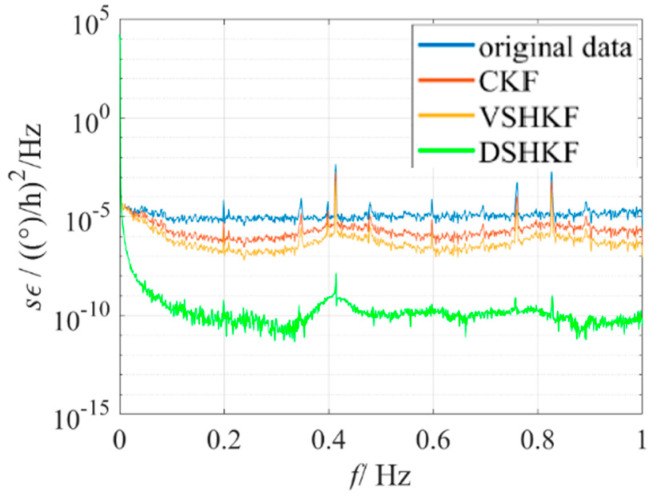
IFOG−C’s PSD at room temperature.

**Figure 11:**
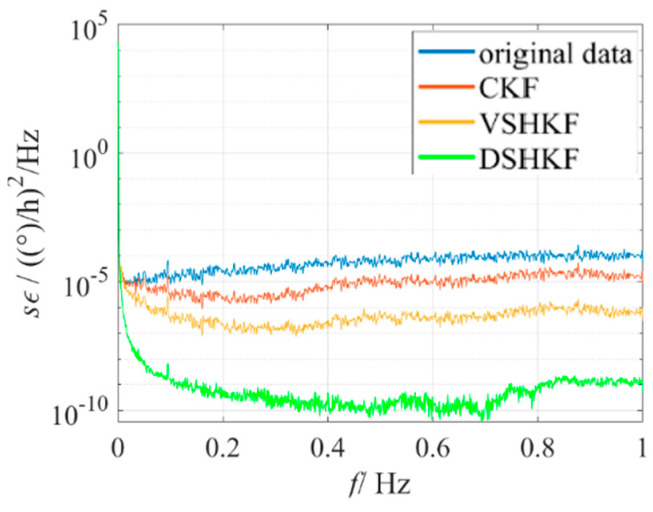
IFOG’s PSD at variable temperature.

As can be seen from Figure 6, like the results of the room temperature experiment, the DSHKF algorithm still has the best filtering effect under dynamics. In order to more intuitively reflect the filtering effect of each algorithm, the filtered IFOG data are subjected to frequency domain Analysis and the power spectrum density is plotted as shown in Figure 8, Figure 9, Figure 10 and Figure 11.

It can be seen from Figure 7, Figure 8, Figure 9 and Figure 10 that the power spectral density distribution of IFOG data filtered by the DSHKF algorithm shows typical colored noise characteristics, and the power spectral density of IFOG data filtered by other algorithms only has some in amplitude. However, the data characteristics of the main component of the angular random walk error (ARW, i.e., angular rate white noise) have not changed significantly. This shows that the DSHKF algorithm can almost completely suppress the main impact of ARW error in IFOG data, while other algorithms cannot achieve this filtering effect.

## 6. Conclusions

This paper proposes a dual adaptive mechanism Kalman filter that automatically switches between variance information and model parameter information to effectively suppress the angular random walk (ARW) error of FOGs. The study implements real-time improvements in Allan variance calculations and incorporates this information into the adaptive Kalman filtering framework. A novel information discrimination mechanism for variance-model adaptive switching was also designed. Compared to traditional adaptive Kalman filters that solely rely on variance information, the proposed filter enhances its ability to make accurate judgments and adaptations based on the FOG’s current data characteristics. This approach avoids filter distortion caused by forcing model errors into measurement errors during adaptation, thereby significantly improving the credibility of the filtering results. The correctness and feasibility of the theoretical framework were verified through comprehensive static and dynamic experiments. These findings not only validate the effectiveness of the proposed method but also provide a new perspective for future research on error suppression in FOGs operating under complex conditions.

## Figures and Tables

**Table 1:** IFOG model parameter estimation results.

	Model Class	Model Coefficient			
		$a_{1}$	$a_{2}$	$a_{3}$	$a_{4}$
IFOG-A	ARIMA(2,1,0)	−0.8833	−0.3729		
IFOG-B	ARIMA(3,1,0)	−0.7750	−0.5631	−0.3534	
IFOG-C	ARIMA(4,1,0)	−0.9486	−0.7320	−0.4756	−0.2171

**Table 2:** Comparison before and after filtering of random walk coefficients in IFOG angle (${}^{\circ}/\sqrt{h}$).

	IFOG-A	IFOG-B	IFOG-C
Original data	$8.5617\times 10^{-5}$	$1.1960\times 10^{-4}$	$7.5021\times 10^{-5}$
After CKF	$4.0276\times 10^{-5}$	$4.1966\times 10^{-5}$	$2.8230\times 10^{-5}$
After VSHKF	$3.4808\times 10^{-5}$	$2.2839\times 10^{-5}$	$1.8899\times 10^{-5}$
After DSHKF	$2.4700\times 10^{-6}$	$3.3563\times 10^{-6}$	$2.8353\times 10^{-6}$

## Data Availability

The raw data supporting the conclusions of this article will be made available by the authors on request.
